# Cellwise outlier detection and biomarker identification in metabolomics based on pairwise log ratios

**DOI:** 10.1002/cem.3182

**Published:** 2019-12-02

**Authors:** Jan Walach, Peter Filzmoser, Štěpán Kouřil, David Friedecký, Tomáš Adam

**Affiliations:** ^1^ Institute of Statistics and Mathematical Methods in Economics TU Wien Vienna Austria; ^2^ Laboratory of Metabolomics, Institute of Molecular and Translational Medicine, Faculty of Medicine and Dentistry Palacký University Olomouc Olomouc Czech Republic; ^3^ Department of Clinical Biochemistry University Hospital Olomouc Olomouc Czech Republic

**Keywords:** biomarker, cell‐rPLR, cellwise outliers, log ratio, metabolomics, robust method

## Abstract

Data outliers can carry very valuable information and might be most informative for the interpretation. Nevertheless, they are often neglected. An algorithm called cellwise outlier diagnostics using robust pairwise log ratios (cell‐rPLR) for the identification of outliers in single cell of a data matrix is proposed. The algorithm is designed for metabolomic data, where due to the size effect, the measured values are not directly comparable. Pairwise log ratios between the variable values form the elemental information for the algorithm, and the aggregation of appropriate outlyingness values results in outlyingness information. A further feature of cell‐rPLR is that it is useful for biomarker identification, particularly in the presence of cellwise outliers. Real data examples and simulation studies underline the good performance of this algorithm in comparison with alternative methods.

## Abstract 2

Data outliers can carry very valuable information and might be most informative for the interpretation. Nevertheless, they are often neglected. An algorithm cell‐rPLR for the identification of outliers in singlecell of a data matrix is proposed. Pairwise log‐ratios between the variablevalues form the elemental information for the algorithm, and the aggregation of appropriate outlyingness values results in outlyingness information. Furthermore,cell‐rPLR is useful for biomarker identification, particularly in the presence of cellwise outliers.

## 1 INTRODUCTION

Metabolomic data, as well as many other data sets from “omics” disciplines, are high dimensional, with many variables and commonly limited by few observations, originating from two or more different groups (controls and diseased). The groups can typically be distinguished at the basis of few variables only, the so‐called biomarkers. Once they are identified, they are important for the interpretation of the group differences.^1^, ^2^

Biomarker identification is getting more challenging if outliers are present in the data.^3^ Outliers in this context can be observations that are somewhat different in their data structure compared with the data majority, and this difference may be caused by measurement problems, different data preprocessing, inconsistencies among the observations, etc.^4^, ^5^ An outlying observation does not necessarily differ in all the variable values, but it could differ just in few variables. This difference could be a data artifact, but it could also refer to a biomarker, for which a difference is to be expected. This means that for the purpose of biomarker identification, outliers could be disturbing if they are related to data artifacts or even helpful otherwise. In the latter case, one would expect that the outliers form a pattern; ie, all observations from that group should have outlying values for the respective biomarker.

**Figure 1:**
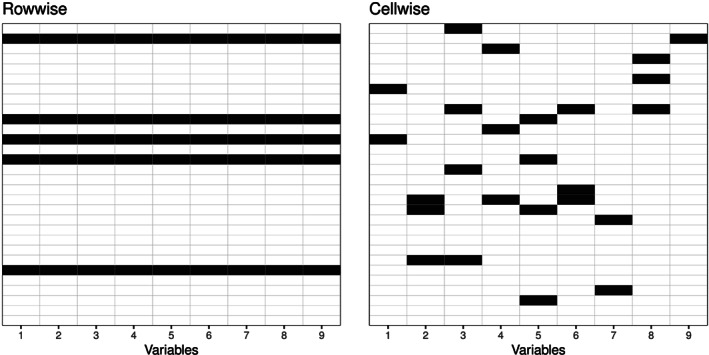
Difference between rowwise (left) and cellwise (right) outliers of a data matrix

Traditionally, outlier identification has been carried out “rowwise,” assuming that the observations are arranged in the rows of the data matrix. This means that if a method identifies an outlier, the complete observation is flagged as such. This situation is visualized in Figure 1 (left), which shows the cells of a data matrix, and the dark cells refer to outliers. Robust statistical estimators would then typically downweight outlying observations.^6^ In contrast to that, Figure 1 (right) refers to a scheme of “cellwise” outliers, where single cell of the data matrix (colored in black) is identified as an outlier. Thus, for each observation, different variables can be outlying. Especially for high‐dimensional data, it might happen that most of the observations will contain at least one cellwise outlier. It would not make much sense to downweight those observations, which contain an outlying cell, since most of the observations would then get downweighted. Cellwise outlier detection is a quite recent topic in robust statistics,^7^ as well as the development of robust estimators with cellwise outliers.^8^ In fact, since our proposed algorithm will be based on variable pairs, there is some similarity to the algorithm of Rousseeuw and Bossche.^7^

Data from metabolomics often have big differences in abundance of individually measured variables. Whereas some variables have low abundances, others are on a much larger scale. This situation is undesirable, since statistical methods could be biased toward higher level abundances. Furthermore, many statistical methods assume that the errors fluctuate around 0 with constant variation. Unfortunately, often with increasing abundances, the variation of noise also increases. Such a situation is called heteroscedasticity. A further important characteristic of metabolomic data is the so‐called size effect. This refers to a situation in which the concentration or abundance is generally different for each sample in the data set, eg, in the analysis of urine samples where concentration strongly depends on the water intake of the patient and other factors. Thus, the obtained data values are not directly comparable, and the data need to be preprocessed first before applying a statistical method.^9^, ^10^ Prepossessing can be done by making use of a specific data transformation^11^ and normalization, eg, the total sum normalization (TSN)^12^ or the probabilistic quotient normalization (PQN).^13^ An alternative is to use the log‐ratio methodology from compositional data analysis, which is based on pairwise log ratios.^14^ Since for two observations, **x** and **y**, and any positive constant *s* (representing the size effect), $\ln\left(\left(s\cdot x\right)/\left(s\cdot y\right)\right)=\ln\left(x/y\right)$, preprocessing is not necessary here.^15^

In this paper, we will introduce an algorithm called cell‐rPLR. The goal of cell‐rPLR is twofold: It can be used for (a) cellwise outlier identification and for (b) biomarker identification. Section 2 describes the theoretical basis of the method. Section 3 introduces a diagnostics plot for cellwise outlier identification. In a simulation scenario, cell‐rPLR is compared with an alternative approach for cellwise outlier detection. Section 4 shows how cell‐rPLR is used for biomarker identification, and the performance is evaluated in scenarios where cellwise outliers are artificially included in the data. Section 5 summarizes the paper, provides information about software, and concludes.

## 2 METHOD

Let us assume a data set arranged in a data matrix **X**, with *n* samples and *d* variables. The matrix **X** consists of elements *x*_*ij*_, where *i*=1,…,*n* and *j*=1,…,*d*. There are *G* ≥ 2 groups of samples in our data, and one can rearrange the samples so that samples belonging to one group are gathered together in one block in **X**. Each block is denoted as **X** ^(*g*)^ with elements $x_{ij}^{\left(g\right)}$ for *i*=1,…,*n*_*g*_, *j*=1,…,*d* and *g*=1,…,*G* and *n*_1_+…+*n*_*g*_=*n*.

The proposed method cell‐rPLR consists of three main steps. In the first step, we use the information of the log ratios between pairs of variables. In the second step, the log ratios are robustly centered and scaled, and an outlyingness function is applied. Finally, the third step projects the data to the original dimensions *n*×*d*. In the following, a detailed description of the individual steps is provided.

### 2.1 Centered and scaled pairwise log ratios

Consider for a pair of variables, with index *j*,*k*∈{1,…,*d*}, the log ratios of their observations:

(1) $$\ln\left(\frac{x_{1j}^{\left(1\right)}}{x_{1k}^{\left(1\right)}}\right),\text{…},\ln\left(\frac{x_{n_{1}j}^{\left(1\right)}}{x_{n_{1}k}^{\left(1\right)}}\right),\ln\left(\frac{x_{n_{1}+1,j}^{\left(2\right)}}{x_{n_{1}+1,k}^{\left(2\right)}}\right),\text{…},\ln\left(\frac{x_{n_{1}+n_{2},j}^{\left(2\right)}}{x_{n_{1}+n_{2},k}^{\left(2\right)}}\right),\text{…},\ln\left(\frac{x_{nj}^{\left(G\right)}}{x_{nk}^{\left(G\right)}}\right).$$

Clearly, the log ratios are 0 if *j*=*k*, and exchanging denominator and nominator leads to the same log ratio but with different sign. Subsequently, we will assign an outlyingness value to each entry of the pairwise log ratios. In order to design an appropriate outlyingness function, the log ratios need to be centered and scaled first. Since potential group differences should not get lost, centering and scaling are performed based on the robust center and scale calculated for the majority group. In case that the group sizes of the biggest groups are equal, one can randomly select one of these biggest groups. For simplicity, suppose now that the first group is the biggest group, thus *n*_1_>*n*_*g*_, for 1<*g* ≤ *G*. Further, we simplify the notation by defining $y_{ijk}:=\ln\left(\frac{x_{ij}^{\left(g\right)}}{x_{ik}^{\left(g\right)}}\right)$, for *i*=1,…,*n*, and *j*,*k*∈{1,…,*d*}. For the following steps, let us drop the indexes *j* and *k* for simplicity, and thus, *y*_*i*_:=*y*_*ijk*_. The log ratios of the first group are the values $y_{1},\text{…}y_{n_{1}}$.

Center and scale of the log ratios of the first group are estimated robustly^6^ as

(2) $${\bar{y}}_{1}=\frac{\sum_{i=1}^{n_{1}}v_{i}y_{i}}{\sum_{i=1}^{n_{1}}v_{i}},$$

where

(3) $$v_{i}=\omega_{c}\left(\frac{y_{i}-\text{median}\left(y_{1},\text{…},y_{n_{1}}\right)}{s_{1}}\right),$$

and $s_{1}=\text{MAD}(y_{1},\text{…},y_{n_{1}})$ is the median absolute deviation, defined as

(4) $$\text{MAD}(y_{1},\text{…},y_{n_{1}})=1.483\cdot {\text{median}}_{i}(|y_{i}-{\text{median}}_{j}(y_{j})|).$$

The function *ω*_*c*_(·) in Equation (3) is Tukey's biweight function,^16^ defined as

(5) $$\omega_{c}(u)={\left(1-{\left(\frac{u}{c}\right)}^{2}\right)}^{2}\cdot I\left(u,c\right),$$

with

(6) $$I\left(u,c\right)=\left\{\begin{array}{ll} \text{1,} & \text{for}\;|u|<c.\\ \;\text{0,} & \text{otherwise}. \end{array}\right.$$

The tuning constant is usually chosen as *c*=4.685. For more details, we refer to Yohai and Zamar^17^ and Maronna and Zamar,^18^ who introduced these concepts in the framework of robust scale estimation.

The robustly centered and scaled values are obtained as

(7) $${\tilde{y}}_{i}=\frac{y_{i}-{\bar{y}}_{1}}{s_{1}}\;\text{for}\;i=1,\text{…},n.$$

Centering and scaling are done for fixed indexes *j*,*k*∈{1,…,*d*}, and now, going back to the notation including these indexes, we end up with robustly centered and scaled values ${\tilde{y}}_{ijk}$. Note that for *j*=*k*, the function arguments in (3) are not defined, because *s*_1_ would be 0. We will thus set the values ${\tilde{y}}_{ijk}:=0$ whenever *j*=*k*. Further, one can see that ${\tilde{y}}_{ijk}=-{\tilde{y}}_{ikj}$, and therefore, it is sufficient to actually compute only the values ${\tilde{y}}_{ijk}$ for *j*<*k*, which saves computational effort.

### 2.2 Outlyingness functions

**Figure 2:**
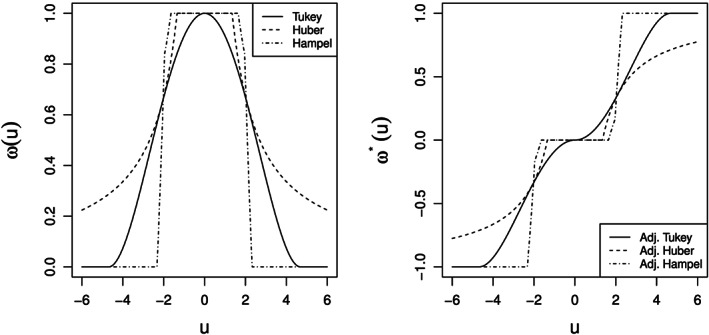
Original (left) and adjusted (right) outlyingness functions

The robustly centered and scaled pairwise log ratios contain information about outlyingness, and this information will be revealed by applying an appropriate outlyingness function to these values. An outlyingness function as proposed in Equation (5) is blue commonly used in robust statistics, eg, in the regression context to downweight (absolute) large residuals, in order to obtain a robust estimator. However, it would not be appropriate for the outlier detection task, since the resulting outlyingness values are in the interval [0,1], and one would lose the sign information of the log ratios. This information will be important, because positive values would refer to a dominance of the nominator and negative values to a dominance of the denominator. Therefore, we propose the adjusted Tukey biweight function as

(8) $$\omega_{c}^{*}(u)=\omega_{c}(u)\cdot sgn(-u)+sgn(u),$$

with the sign function

(9) $$sgn(v)=\left\{\begin{array}{ll} \text{1,} & \text{for}\;v\geq 0,\\ -, & \text{otherwise}, \end{array}\right.$$

yielding values in [−1,1]. Figure 2 shows the original definition of the Tukey biweight weights (left plot) and compared with the adjusted version leading to outlyingness values (right plot).

The adjusted outlyingness function is applied to the robustly centered and scaled pairwise log ratios from (7). Outlyingness values around 0 represent nonoutliers, and values closer to −1 or +1 represent potential outliers.

The shape of the outlyingness function will determine the characteristics of the outlier detection method, and thus, other outlyingness functions shall be considered as well. In the literature on robust statistics, many proposals are available, such as Huber's^4^ and Hampel's^19^ functions. Below, we will propose the adjusted versions, resulting in outlyingness values in the interval [−1,1].

Huber function: The original definition is

(10) $$\omega_{k}\left(u\right)=min\left(1,\frac{k}{\left|u\right|}\right),$$

where *k* is a tuning parameter, typically taken as 1.345.^4^ The assignment of 1 to a broader range of values improves the efficiency of robust estimators, while still keeping their robust properties. The adjusted version

(11) $$\omega_{k}^{*}\left(u\right)=\left(\omega_{k}(u)-1\right)\cdot sgn(-u),$$

assigns outlyingness values of 0 to those (nonoutlying) log ratios, which are still in the usual range. The resulting shape of the adjusted Huber function can be seen in Figure 2 (right); the left plot shows the original definition.

Hampel function: The original definition is

(12) $$\omega_{h}\left(u\right)=\left\{\begin{array}{ll} 1 & \left|u\right|\leq c_{1}\\ \frac{c_{1}}{\left|u\right|} & c_{1}\leq \left|u\right|\leq c_{2}\\ \frac{c_{3}-\left|u\right|}{c_{3}-c_{2}}\frac{c_{1}}{\left|u\right|} & c_{2}\leq \left|u\right|\leq c_{3}\\ 0 & c_{3}<\left|u\right| \end{array}\right.,$$

where the tuning parameters are typically chosen as *c*_1_=*z*_0.95_, *c*_2_=*z*_0.975_, and *c*_3_=*z*_0.99_, with *z*_*q*_ as the *q* quantile of the standard normal distribution. The Huber function approaches 0 asymptotically, whereas in the Hampel function, one obtains outlyingness values of 0 according to the tuning parameter *c*_3_. The adjusted version of the Hampel function is

(13) $$\omega_{h}^{*}\left(u\right)=\left(\omega_{h}(u)-1\right)\cdot sgn(-u),$$

which again provides values in [−1,1]. Figure 2 shows the original (left) and adjusted (right) Hampel function.

### 2.3 Aggregation of outlyingness values

Let us denote the adjusted outlyingness function by *ω* ^*^(·), which refers to one of the proposed functions in Section 2.2. We apply this function to the centered and scaled pairwise log ratios (see Section 2.1), resulting in outlyingness values

(14) $$w_{ijk}^{*}=\omega^{*}({\tilde{y}}_{ijk}),$$

for *i*=1,…,*n* and *j*,*k*∈{1,…,*d*}. These outlyingness values are stored in an array **W** ^*^ with *n* rows, *d* columns, and *d* slices.

Since we aim at a method for cellwise outlier detection, the outlyingness values in the array **W** ^*^ need to be aggregated appropriately in order to obtain outlyingness values for each cell in the *n*×*d* data matrix **X**. For robustness reasons, we propose the aggregation into outlyingness values

(15) $$w_{ij}=\text{median}\left(w_{ij1}^{*},w_{ij2}^{*},\text{…},w_{ijd}^{*}\right),$$

for *i*=1,…,*n* and *j*=1,…,*d*, and they are collected in the *n*×*d* outlyingness value matrix **W**. Note that it would also be possible to aggregate the outlyingness values $w_{ijk}^{*}$ according to the second index. This would result in the same values of aggregated outlyingness values but with reverse sign, because the considered outlyingness functions have the property *ω* ^*^(*u*)=−*ω* ^*^(−*u*) and because ${\tilde{y}}_{ijk}=-{\tilde{y}}_{ikj}$.

### 2.4 Cell‐rPLR algorithm for outlier diagnostics

The algorithm for cell‐rPLR can be summarized as follows:

Step 1: Compute all pairwise log ratios $\ln\left(\frac{x_{ij}^{\left(g\right)}}{x_{ik}^{\left(g\right)}}\right)$ for *i*=1,…,*n* and *j*,*k*∈{1…,*d*} with *j*>*k* (see 1).
Step 2: Center and scale them robustly according to the majority group. This gives values ${\tilde{y}}_{ijk}$, for all *i*,*j*,*k*.
Step 3: Apply an outlyingness function to ${\tilde{y}}_{ijk}$, which yields outlyingness values $w_{ijk}^{*}$ (see 14).
Step 4: Aggregate the outlyingness values according to (15) to obtain the final outlyingness values *w*_*ij*_, arranged in the outlyingness values matrix **W**.

If the weighing function is monotone, the change of order of steps 3 and 4 yields the same results.

Note that this outlier detection algorithm is supervised, because the group information of the observations is used in step 2.

We do not specify an outlier cutoff value for identifying outlying cells. Rather, we visualize the information contained in **W**, by using different colors for positive (red) and negative (blue) values and different color intensity, with light color for outlyingness values close to 0 and intense color otherwise. Thus, cell‐rPLR serves as a visual outlier diagnostics tool. Note that the intensity of the color strongly depends on the chosen outlyingness function. Furthermore, one should keep in mind that the outliers are analyzed in comparison with one group (eg, the majority group) and need to be interpreted this way.

### 2.5 Cell‐rPLR algorithm for biomarker identification

As noted above, the cell‐rPLR can also be used for feature selection. In this case, however, this is limited only to the case of *G*=2 groups. The outlyingness values *w*_*ij*_ from step 4 of the algorithm are arranged groupwise, and we have outlyingness values $w_{j}^{\left(1\right)}=\{w_{1j},\text{…},w_{n_{1}j}\}$ for the first group and outlyingness values $w_{j}^{\left(2\right)}=\{w_{n_{1}+1,j},\text{…},w_{nj}\}$ for the second group, for *j*=1,…,*d*. For feature selection, we compare the medians in both sets of outlyingness values by

(16) $$m_{j}=\left|\;\text{median}\left(w_{j}^{\left(1\right)}\right)-\text{median}\left(w_{j}^{\left(2\right)}\right)\;\right|.$$

The larger the difference is, the more important the variable is for the discrimination of the groups. Note that the size of *m*_*j*_ for different *j* can indeed be compared, since the outlyingness values are on the same scale. One can either sort the values *m*_*j*_ in descending order or obtain a ranked variable list, with potential biomarkers at the beginning of the list. On the other hand, it might be desirable to obtain a cutoff value indicating potential biomarkers. Therefore, we will make use of a permutation test. Permutation tests^20^, ^21^ are wildly used for significance testing. They are based on resampling and try to estimate the distribution of the test statistic. The goal is to estimate a *P* value for the testing problem. In our case, the null hypothesis states that there is no difference between the two groups in the data for a certain variable, ie, *m*_*j*_=0, and thus, the variable is not a biomarker.

The permutation tests for cell‐rPLR can be described as follows:

Step 1: Use as an input the matrix **W** ^*^ with the elements $w_{ijk}^{*}$, defined in (14).
Step 2: Randomly permute the values $w_{ijk}^{*}$ according to the index *j*, resulting in values $w_{ijk}^{*}(b)$ for replication *b*∈{1,…,*B*}.
Step 3: Aggregate the values from the *b*th permutation as in Equation (15), yielding values *w*_*ij*_(*b*).
Step 4: Compute the differences according to Equation (16), resulting in *m*_*j*_(*b*), for *j*=1,…,*d*.
Step 5: Compute the proportion
  $$\frac{1}{B}\overset{B}{\underset{b=1}{\sum }}\left(m_{j}\leq m_{j}(b)\right),$$

for *j*=1,…,*d*, which is interpreted as *P* value for the *j*th variable. Here, *m*_*j*_ refers to the values from (16) for the unpermuted data.

In our numerical experiments, we used *B*=1000. The computations are still feasible, because the input matrix **W** ^*^ is fixed and with the *B* permutations, the *P* values for all variables are returned.

The permutations carried out in step 2 are done with respect to the index *j* only, which is numerically easy and quick to do. An alternative would be permuting all the elements in the blocks of the array **W** ^*^. Technically, this would be more difficult and time‐consuming, and the results would essentially be the same. The reason is because a permutation only in index *j* already destroys the group structure, since the original outlyingness values are based on values ${\tilde{y}}_{ijk}$, which are centered and scaled according to one of the groups. The median aggregation in (15) carries on this destroyed group structure to the test statistic (16).

Note that an alternative for the permutation test would be to start the permutations directly from the values ${\tilde{y}}_{ijk}$, ie, by permuting the log ratios already. This would be computationally far too expensive, at least for high‐dimensional data, and again, the results will be very similar to our proposed version.

## 3 PERFORMANCE OF CELL‐RPLR FOR OUTLIER IDENTIFICATION

The cell‐rPLR algorithm results in outlyingness values *w*_*ij*_, arranged in the outlyingness values matrix **W**; see Equation (15), which indicates cellwise outlyingness, and they can be visualized in a heatmap. A heatmap is a graphical visualization of the cells of a matrix, with the corresponding number of rows and columns, where each value of the data matrix is represented by a color information. We will use red color for positive outlyingness values and blue color for negative values, and the magnitude of the outlyingness value will determine the color saturation (values around 0 in light colors).

### 3.1 Data sets

Three metabolomic data sets were used to demonstrate the usefulness of the method. The data sets differ in size and in the number of groups. For the last two data sets, expert knowledge about biomarkers is available.

IMD: This data set^22^ consists of plasma samples from infants (50 control samples and 16 samples) with different metabolic diseases analyzed in the Laboratory of Metabolomics, Institute of Molecular and Translational Medicine, Faculty of Medicine and Dentistry, Palacký University Olomouc, Czech Republic. There are in total four different inherited metabolic disorders (IMD)—phenylketonuria (PKU), homocystinuria (HCYS), methylmalonic aciduria (MMA), and propionic aciduria (PA), each with different number of samples varying from 2 to 6. The samples were analyzed using the AbsoluteIDQ p150 kit (BIOCRATES Life Sciences AG, Austria). All the measurements were performed on a QTRAP 5500 (AB SCIEX, USA; flow injection analysis, ESI in both + and − MRM mode), and the data were processed in MetIQ software (AbsoluteIDQ kit). In total, 163 metabolites were quantified.
MTBL59: This data set is described in Franceschi et al and Wehrens et al^23^, ^24^ and can be downloaded from the MetaboLight web page https://www.ebi.ac.uk/metabolights/MTBLS59. It contains 20 apple samples, which were analyzed by liquid chromatography–mass spectrometry (LC‐MS). The first 10 samples of apples were analyzed without any modification. The second 10 samples were spiked with naturally occurring substances in apples. In that way, two groups with five known biomarkers were created and can be analyzed. Data preprocessing was carried out as outlined in Wehrens et al.^24^ Only the first 9 minutes of the chromatography was subtracted, leading to 197 features.
PKU: This data set concerns plasma samples from PKU patients (*n*_1_ = 27) and healthy controls (*n*_2_ = 17), where untargeted metabolomics analysis based on the work of Wang et al^25^ was performed in the Laboratory of Metabolomics, Institute of Molecular and Translational Medicine, Palacký University Olomouc, Czech Republic. The data were processed using the vendor software Compound Discoverer 3.0 (Thermo Fisher Scientific) and exported to the R software to perform correlation analysis to merge redundant features (RT difference ≤ 0.02 min, r ≥ 0.95, centered log ratio [clr] transformation) and to perform other statistical evaluation (the data were corrected using QC samples and LOESS regression^26^; potential metabolites with CV higher than 30% were excluded from further data processing). There are in total 2336 features in the data set. On the basis of the biological analysis of the PKU disease, there are four known biomarkers.^27^, ^28^, ^29^

### 3.2 Visualization of cellwise outliers

**Figure 3:**
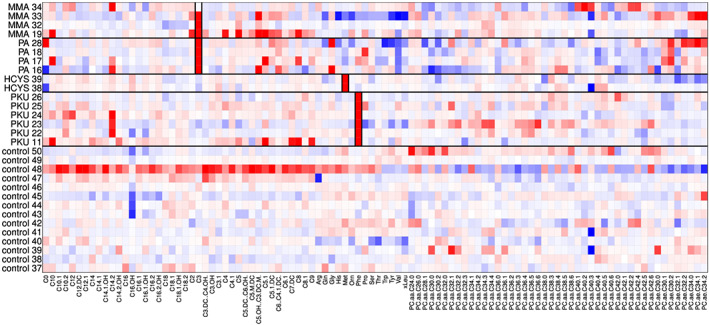
Outlier diagnostics for the IMD data, using the adjusted Tukey biweight function

**Figure 4:**
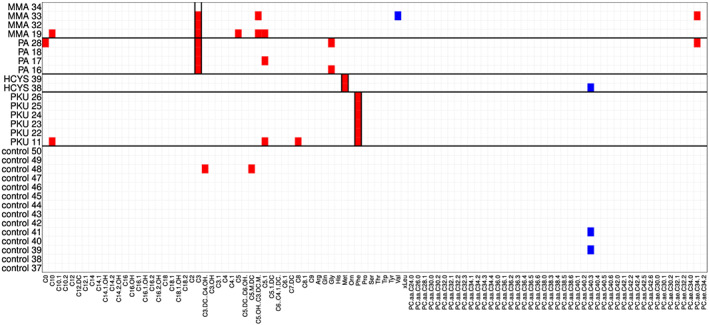
Outlier diagnostics for the IMD data, using the adjusted Hampel function

We use the data set IMD to visualize cellwise outliers. In this multigroup data set, centering and scaling were performed according to the majority group, which is the control group; see Equation (7). Then, the cell‐rPLR method was applied as described in Section 2. The resulting heatmap is shown in Figure 3 using the adjusted Tukey biweight function (8) and in Figure 4, which is based on the adjusted Hampel function (12). For reasons of space, we omitted the first 36 control patients from the visualization and showed only controls 37 to 50 and the patients from the different disease groups, separated by black horizontal lines. At a first glance, Figure 4 seems to provide a much clearer picture concerning potential cellwise outliers. This is due to the fact that the adjusted Hampel function assigns 0 to a much broader range of “normal” values than the adjusted Tukey biweight function. A red value corresponds to a “positive outlier” and to a value that is higher than expected. A blue value indicates a “negative outlier,” with a value lower than expected. Figure 3 shows that control observation 48 has systematic bias from the others: The first block of variables for this sample is outlying in the positive direction, whereas the last block is outlying in the negative direction. This is not so clearly visible in Figure 4. If an entire signal of a sample is outlying, the sample preparation could have been done a bit different from the other samples, a different device setting could have been used during the analysis, or the control might have been biased by a certain unknown nonphysiological state. Moreover, variable *PC*.*aa*.*C*40.3 shows several negative outliers. However, the most visible (positive) outliers are in vertical blocks, indicated by the black rectangulars. In fact, these rectangular regions are the known metabolites *C*3 for group MMA and PA, *Met* for HCYS, and *Phe* for PKU. The first cell (patient MMA 34) of the biomarker *C*3 was not identified as outlier, which might mean that this patient is in an early stage of his/her disease or is possibly already cured. Note that positive outliers (red color) mean that the corresponding variables have increased values (dominance) for these observations.

This example shows that the choice of the outlyingness function should be based on the analysis goal. If a clear indication of outlyingness is desired, the adjusted Hampel or Huber function could be used, where Hampel would in general lead to more saturated colors. The adjusted Tukey biweight function would give more light red/blue color instead of white cells but indicate clearer small deviations from the “normal” behavior.

### 3.3 Simulation study for outlier identification

In this section, we will more thoroughly test the algorithm cell‐rPLR for outlier detection. This will be based on the data sets MTBL59 and PKU, where (additional) cellwise outliers will be generated by simulations, using

(17) $${\tilde{x}}_{ij}=x_{ij}\cdot M+A,$$

where a multiplicative effect *M* is generated from a uniform distribution $U\left(0.2,0.5\right)$ or $U\left(2,10\right)$ (with equal probability) and an additive effect *A* is generated from $U\left(2,5\right)$. This modification is done for a random percentage between 5% and 15% of the cells, which are randomly picked.

The modified cells are treated as “true” outliers, which should be identified with the algorithm. Note that by accident, scheme (17) could produce values that are still not very extreme and thus hard to identify as outliers. On the other hand, the data sets could already include cellwise outliers, but since they are unknown, an identification by the algorithm would count as a wrong decision.

We will compare our algorithm with the method detect deviating cells (DDC),^7^ which can be considered as a state‐of‐the‐art cellwise outlier identification method. In the first step, DDC robustly standardize the columns of the data matrix, univariate outlier detection is applied to all variables separately, and outlying cells are marked. Later, the correlation structure is computed based on the nonmarked observations. This is followed by the prediction of each nonmarked data cell in the same row, considering only the correlating variables. The final outlyingness level is determined by the difference between the predicted and the reported values for each cell. The bigger the difference, the more outlying is the cell. In order to apply DDC appropriately, we first preprocess the data with the PQN transformation. The original version of DDC algorithm does not use the data label, and this version is also used in our simulations. However, the DDC could be redesigned in order to incorporate the group information.

**Figure 5:**
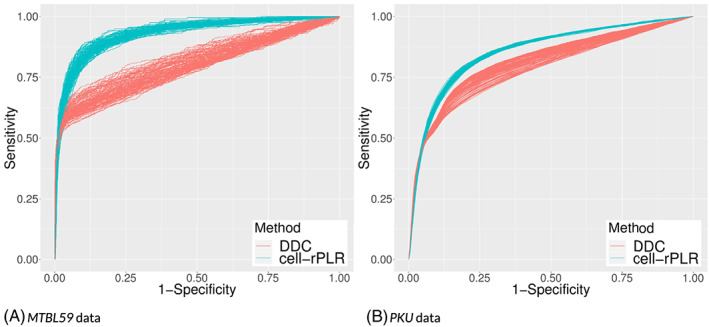
A,B, Receiver operating characteristic (ROC) curve for the identification of cellwise outliers of the two algorithms: detect deviating cells (DDC) (red) and cellwise outlier diagnostics using robust pairwise log ratios (cell‐rPLR) (blue)

In each of the 100 iterations, the receiver operating characteristic (ROC) curve was computed. The ROC curve shows the proportions of correctly identified outliers (sensitivity) and incorrectly identified nonoutliers (one minus specificity) for varying outlier cutoff points. Figure 5 shows the 100 different ROC curves for the cell‐rPLR (with the adjusted Tukey biweight function) and the DDC algorithm for the data sets (a) MTBL59 and (b) PKU. A good method would lead to an ROC curve, which is close to the upper left corner of the plot (all outliers correctly identified, no false outlier indication). The plots reveal that the performance of the algorithms is better for the MTBL59 data set (197 variables) than for the PKU data set with much more variables (2336). In both data sets, the cell‐rPLR algorithm clearly shows a better performance compared with the DDC method. Note, however, that the DDC method does not make use of the grouping information.

## 4 PERFORMANCE OF CELL‐RPLR FOR BIOMARKER IDENTIFICATION

As in the previous Section 3.3, we use the data sets MTBL59 and PKU to test the performance of the method cell‐rPLR for identifying biomarkers. Note that these data sets only consist of two groups of observations, which is the setting we require for cell‐rPLR biomarker identification. In particular, we are interested in the behavior of the method in the presence of contamination by cellwise outliers. Therefore, the same kind of data contamination is applied as introduced in (17), and for 100 simulation runs, 5*%*, 10*%*, 15*%*, 20*%*, and 25*%* of randomly selected cells of the data matrices are contaminated.

We will compare the following methods:

cell‐rPLR: This algorithm is applied as described in Section 2.5. A ranked list for the variable importance is obtained by the comparison of the medians of the outlyingness values; see Equation (16). For a list of identified biomarkers, the permutation test as described in Section 2.5 is applied.
PLS‐VIP: Partial least squares discriminant analysis (PLS‐DA)^30^, ^31^, ^32^ is applied to the data set, which is doing PLS regression on the binary response containing the group labels. This method results in a projection of the samples on few latent variables. Since PLS‐DA does not return the information of the most important variables for group discrimination, a variable importance projection (VIP) score^33^, ^34^ is computed, which sums up the contribution of each variable in the model. The VIP scores are then sorted in ascending order so that a ranking of the variables is created. It is generally accepted that a variable should be marked as a biomarker if its VIP score is bigger than 1.^35^, ^36^
PLS‐SR: There are many methods available for extracting the importance of the variables in a PLS‐DA model.^37^ Here, we consider the selectivity ratio (SR),^38^, ^39^ which is using scores and loadings from PLS‐DA and computes a proportion of explained variance for each variable. Again, this results in a list of variables, sorted according to their importance, as well as in a list of identified biomarkers, which are selected as variables with SR score above the 0.95 quantile of the distribution *F*_*n*−2,*n*−3_. 40
PRM‐VIP: Partial least squares discriminant analysis is not robust against data outliers,^41^ and thus, the robust counterpart based on partial robust M‐regression (PRM)^42^ is used. This is followed by computing the VIP score as a measure for variable importance.
PRM‐SR: As before, PRM is applied but followed by computing the SR.
DesEq: This method builds on two steps: (a) an internal normalization of the variables by their geometric means and (b) a decision about the importance of variables.^43^ For this purpose, a negative binomial generalized linear model is fit to each variable, and the *P* value from a Wald test^44^, ^45^ is computed for creating a rank of importance for the variables and a list of identified biomarkers. The method removes outliers based on Cook's distance.
Aldex: This method is based on Monte Carlo simulations of the Dirichlet multinomial model.^46^, ^47^ The clr transformation^48^ is internally used. Then, *P* values obtained from Welsch's test^49^ are employed for ranking the variables and for returning a list of identified biomarkers.

For the methods employing PLS or PRM, a preprocessing step is necessary, and we decided to apply the PQN transformation,^13^ which is widely used.

**Figure 6:**
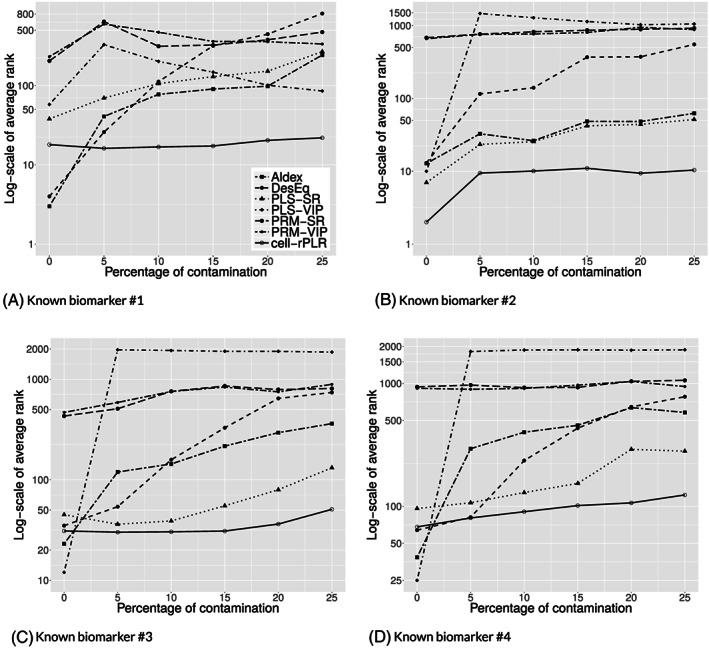
A‐D, Average ranks of the methods for the identification of the four known biomarkers in the PKU data, in a simulation setting with increasing amount of contamination

**Figure 7:**
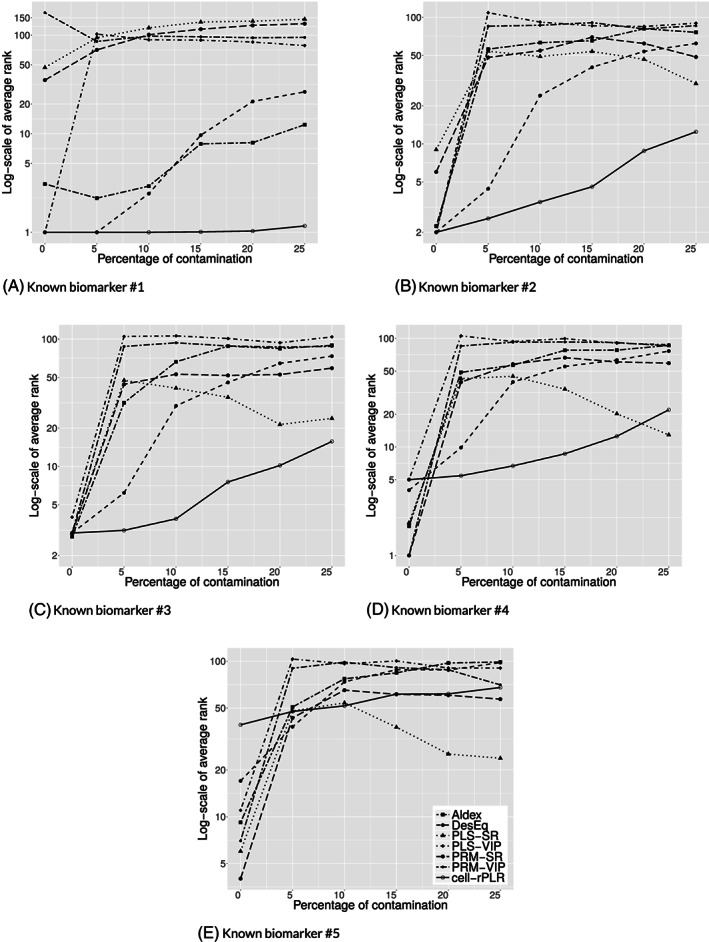
A‐E, Average ranks of the methods for the identification of the five known biomarkers in the MTBL59 data, in a simulation setting with increasing amount of contamination

Figures 6 and 7 show the results of the simulation study. The subplots in these figures report the resulting ranks for one particular known biomarker. Figure 6 refers to the results for the PKU data with four known biomarkers, and Figure 7 to those from the MBTL59 data with five known biomarkers. We report here the average ranks (over the 100 simulations) for the individual methods. Because of the large number of variables, the average ranks are shown on a base‐10 logarithmic scale.

Figures 6 and 7 show that for many of the methods, the average ranks for the known biomarkers increase with increasing data contamination. An exception is the cell‐rPLR algorithm, which leads in general to the lowest ranks (at least for the contaminated situation) and also to stability in case of contamination. In particular for the PKU data (Figure 6), the PRM‐based methods show a very poor performance, even for the uncontaminated data. This is because in this very high‐dimensional data set, existing cellwise outliers might affect several observations and PRM then downweights all these observations. Especially when adding cellwise outliers, PRM leads to poor results exactly because of this reason; see also Figure 7. Depending on the specific biomarker, Aldex and DesEq also lead to reasonable performance, but they are clearly affected by the contamination. For the PKU data (Figure 6), PLS‐SR is also quite competitive, but it completely fails for the PKU data.

**Figure 8:**
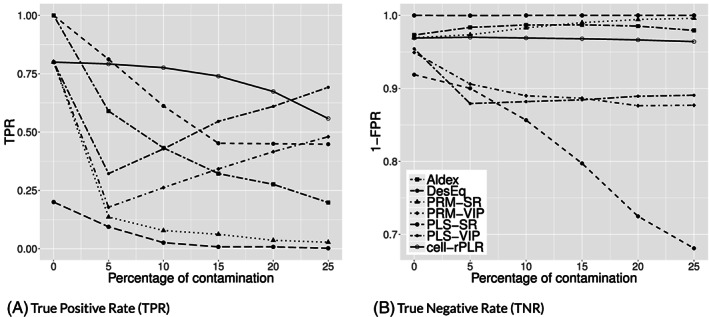
A, B, Performance of the methods for their ability in biomarker identification for the MTBL59 data set, for different levels of cellwise contamination

Each of the considered methods returns the information if a variable is identified as a biomarker or not. In case of cell‐rPLR, the permutation test (see Section 2) is employed to deliver this information. Thus, we evaluate the performance of correct biomarker identification based on the same simulation scenario as used before, for the data sets PKU and MTBL59. For each method, the true positive rate (TPR) as the proportion of correctly identified biomarkers (Sensitivity) and the true negative rate (TNR) as the proportion of correctly identified nonbiomarkers (Specificity) are computed. In the ideal case, both the TPR and TNR should yield values close to 1. Figure 8 summarizes the average values for TPR and TNR over the 100 simulation runs and for the MTBL59 data set. According to the false positive rate (FPR), the methods PLS‐SR, Aldex, PRM‐SR, and cell‐rPLR show excellent behavior. However, PLS‐SR has a very poor TPR (true biomarkers not identified). Also, the TPR of PRM‐SR suffers from the contamination, because PRM can only cope with rowwise contamination. Aldex and DexEq are also sensitive to contamination, even the algorithm cell‐rPLR shows a slight deterioration with increasing contamination—probably due to some effect of the permutation test. Overall, however, cell‐rPLR is the clear winner under contamination but shows also competitive performance without contamination.

## 5 SUMMARY AND CONCLUSIONS

This paper introduced a method called cell‐rPLR, which allows to detect outlying cells in a data matrix of metabolomic data and also identifies biomarkers, even in presence of such cellwise outliers. This method does not require the usual data preprocessing (normalization and scaling) needed by many other methods, because the elemental information is pairwise log ratios between the variable values. The size effect that is often present in metabolomic data thus is automatically filtered out by using the log ratios. To the best of our knowledge, this is the first paper focusing on cellwise outliers in the context of metabolomic data. Cell‐rPLR applies an outlyingness function to the robustly centered and scaled pairwise log ratios, and the results are outlyingness values for each observation and for each pair of variables (three‐way array). After robust aggregation, one obtains an outlyingness values matrix of the same dimension as the data matrix, containing the cellwise outlyingness information. The outlyingness values are in the interval [−1,1]; outlyingness values around 0 point at “normal” data cells, and outlyingness values close to +1 or −1 indicate atypical data cells. This information can be visualized in a heatmap by using a color coding of the outlyingness values. The appearance of the heatmap depends a lot on the chosen outlyingness function, but the choice of the outlyingness function would not essentially change a resulting ROC curve (this is valid at least for the outlyingness functions proposed in this paper). Thus, the heatmap can be regarded as a diagnostics tool for investigating the data structure. Cellwise outliers could indicate data problems, but they also indicate biomarkers if they are systematically present in one variable of a particular data group. A permutation test for biomarker identification was developed based on the cell‐rPLR algorithm. On the basis of the simulations using artificially contaminated real data, the performance of cell‐rPLR was compared with a state‐of‐the art cellwise outlier detection algorithm, where it turned out that cell‐rPLR was more accurate and at the same time did spot fewer “normal” data cells as outliers. Similarly, simulations have shown that the performance of cell‐rPLR in biomarker identification was competitive to alternative methods and superior in the presence of cellwise contamination.

The cell‐rPLR is implemented in the software environment R 50 and can be downloaded as package cellrPLR from https://github.com/walachja/cellrPLR. The package contains the implementation of the cell‐rPLR algorithm and the permutation test for biomarker identification. Heatmaps for visualizing cellwise outliers are included as well. Furthermore, for an easier exploration and understanding of the data, a Shiny app is a part of the package. Shiny,^51^ an open source R package, is a web application and serves as an interactive tool for visualization. The Shiny app allows to interactively apply different outlyingness and aggregation functions for the cell‐rPLR algorithm and supports zooming into regions of the data matrix to see more details. Moreover, the variables can be interactively ordered based on their importance for the group discrimination.

In our future work, we will focus on an automatic classification of cellwise outliers into technical artifacts (cellwise outliers) and biological artifacts (biomarkers) using the cell‐rPLR algorithm. This is particularly important in case of very small groups of data, where this distinction is difficult with the current version of the algorithm. Moreover, the cell‐rPLR method will be extended to biomarker identification for the multigroup case.
